# LRIG1 and epidermal growth factor receptor in renal cell carcinoma: a quantitative RT–PCR and immunohistochemical analysis

**DOI:** 10.1038/sj.bjc.6601208

**Published:** 2003-09-30

**Authors:** M Thomasson, H Hedman, D Guo, B Ljungberg, R Henriksson

**Affiliations:** 1 Department of Radiation Sciences, Oncology, Umeå University Hospital, Umeå, Sweden; 2 Department of Surgical and Perioperative Sciences, Urology, Umeå University Hospital, Umeå, Sweden; 3 AstraZeneca, Medical Department, Mölndal, Sweden

**Keywords:** RCC, EGFR, kidney tumour, LIG-1

## Abstract

In all, 31 renal cell carcinomas (RCCs) were examined for expression of the potential tumour suppressor LRIG1 (formerly Lig-1) and the epidermal growth factor receptor (EGFR). Eight matched samples of uninvolved kidney cortex were also evaluated. Gene expression was examined by quantitative real-time RT–PCR. In the eight matched sample pairs (uninvolved kidney cortex and tumour), protein expression was examined by immunohistochemistry. Conventional (clear cell) tumours showed an expected upregulation of EGFR. LRIG1 expression was generally downregulated in conventional and papillary RCC but not in chromophobic RCC. The ratio between EGFR and LRIG1 was more than 2.5-fold higher in the eight tumours compared with matched uninvolved kidney cortex and was at least two-fold higher than the mean normal ratio in 21 of 31 samples analysed. The observed downregulation of LRIG1 and increased EGFR/LRIG1 ratios are consistent with LRIG1 being a suppressor of oncogenesis in RCC by counteracting the tumour-promoting properties of EGFR. Further studies are justified to elucidate the explicit role of LRIG1 in the oncogenesis of RCC.

Renal cell carcinoma (RCC) represents 2–3% of all cancer. There are distinct subclasses of RCC, differing in histopathological appearance and genetic alterations. The most common accounting for approximately 70% of these tumours are conventional (clear cell) RCC. Papillary RCC accounts for 10–15%, chromophobe RCC for about 5% and collecting duct carcinoma for less than 1% (Kovacs *et al*, 1997; Störkel *et al*, 1997). Despite many efforts to develop effective therapies, including immunotherapy, hormone manipulation and chemotherapy, the survival of advanced RCC still remains poor (Henriksson *et al*, 1998; Motzer and Russo, 2000). So far, the knowledge of the different histopathological entities of RCC has not led to differences in the management of RCC. Previous studies have shown epidermal growth factor receptor (EGFR) overexpression in RCC, but its prognostic significance is controversial (Lager *et al*, 1994; Ljungberg *et al*, 1994; Yoshida and Tosaka, 1994; Hofmockel *et al*, 1997). The EGFR ligands EGF and TGFα are also frequently overexpressed in RCC. TGFα has a low expression in normal kidney and may be involved in an autocrine loop with EGFR in RCC (Mydlo *et al*, 1989). This is particularly interesting since therapies targeting EGFR have shown interesting results in the treatment of other cancers (Baselga and Averbuch, 2000; Mendelsohn and Baselga, 2000). Human LRIG1 (formerly LIG1) has recently been cloned and characterised (Nilsson *et al*, 2001). LRIG1 is a transmembrane cell surface protein with structural similarities to the *Drosophila* (fruit fly) cell surface protein Kekkon-1 that participates in an EGF-driven negative feedback loop (Ghiglione *et al*, 1999). *LRIG1* is localised to chromosome 3p14 (Nilsson *et al*, 2001), a region often deleted in conventional RCC (van den Berg and Buys, 1997). Owing to its interesting chromosomal localisation and its potentially EGFR-inhibiting qualities, we found it of interest to examine the expression of LRIG1 in RCC and to evaluate its relationship with the expression of EGFR, in RCC and in normal kidney tissue from the same patients.

## MATERIALS AND METHODS

### Patients

Specimens and clinical data were obtained from 31 patients with RCC who underwent nephrectomy at the Umeå University Hospital between 1986 and 1998 (Table 1

**Table 1:** Characterisation of patients and tumours included in the study

Total no of patients	31
Sex (male/female)	17/14
Age (years) median (range)	64 (36–85)
Tumour diameter, median (range)	80 (30–250)
TNM stage 1	7
TNM stage 2	5
TNM stage 3	10
TNM stage 4	9
Tumour grade 2	5
Tumour grade 3	21
Tumour grade 4	5
Known metastasis at the time of nephrectomy	9
Survival time (months), median (range)	44 (1–139)

), in accordance with the regulations approved by the ethical committee of the Umeå University Hospital. Of these tumours, 18 were conventional (clear cell), 10 papillary and three chromophobe RCCs. In eight (five with conventional and three with papillary RCC) of the patients, we obtained matched specimen of grossly non-neoplastic kidney cortex tissue.

### Quantitative RNA analysis

RNA was prepared from tissue samples by mechanical disruption in TRIzol reagent (Gibco-BRL, Rockville, MD, USA), followed by chloroform extraction and alcohol precipitation according to the manufacturer's instructions as described (Nilsson *et al*, 2001). Real-time quantitative reverse transcription (RT)–PCR was performed as described (Nilsson *et al*, 2001) using an iCycler (Bio-Rad, Hercules, CA, USA). Primers and probes (Table 2

**Table 2:** Oligonucleotide primer and probe sequences for real-time quantitative RT-PCR

**Templates**	**Oligonucleotides**	**Sequences**	**PCR product size (bp)**
EGFR	Forward primer	5′-TCCCTCAGCCACCCATATGTAC-3′	125
	Reverse primer	5′-GTCTCGGGCCATTTTGGAGAATCC-3′	
	Probe	5′-afATCACC^b^QCACGGAACTTTGGGCGACTATCTGCGTC-3′	
LRIG1	Forward primer	5′-GGTGAGCCTGGCCTTATGTGAATA-3′	108
	Reverse primer	5′-CACCACCATCCTGCACCTCC-3′	
	Probe	5′-afACACAT^b^QGGTAGCGGCCCTCGTGCCCG-3′	
18S	Forward primer	5′-CGGCGACGACCCATTCGAAC-3′	99
	Reverse primer	5′-GAATCGAACCCTGATTCCCCGTC-3′	
	Probe	5′-afCCTATCAAC^b^QTTCGATGGTAGTCGCCGTGCC-3′	

a *Note.* f denotes a 5′-conjugated fluorescein and

b Q denotes a T with a conjugated Dark Quencher molecule.

) were synthesised by Scandinavian Gene Synthesis AB (Köping, Sweden). RNA samples were run in triplicate using 20 ng of RNA per reaction. Relative quantification was performed by comparing the threshold cycle values (Ct) for the samples, with standard curves generated with cloned cDNAs of respective genes (Nilsson *et al*, 2001). To correct for differences in RNA quality and quantity, the apparent levels of 18S rRNA were used to normalise the EGFR and LRIG1 values in respective RNA samples.

### Immunohistochemistry

From blocks of formalin-fixed and paraffin-embedded tissues, 5 *μ*m-thick sections were cut and mounted with samples of normal and tumour tissue on the same slide. The sections were deparaffinised by standard methods. Endogenous peroxidase activity was blocked with 3% peroxidase in methanol for 20 min and washed in PBS 3 × 5 min. The samples were merged in 0.01 M citrate buffer pH 7.3 and treated with microwaves (1400 W 1 × 5 min LRIG1 and 720 W 1 × 5 min+500 W 2 × 5 min for EGFR). Samples, preincubated with 10% goat serum for 30 min to block unspecific binding, were incubated with primary antibodies (LRIG1, a polyclonal rabbit antibody, diluted 1 : 500 and for EGFR a polyclonal rabbit antibody, Santa Cruz Biotechnology, Santa Cruz, CA, USA diluted 1 : 100, overnight). Sections were washed in PBS 3 × 5 min, before application of the biotinylated secondary goat antibody (Vector Laboratories, Burlingame, CA, USA) diluted 1 : 200 for 40 and 30 min, respectively. Thereafter, specimens were washed 3 × 5 min in PBS and incubated with the DAB substrate kit (Vector Laboratories), according to the manufacturer's instructions. The sections were rinsed in water, counterstained with Mayer staining, again rinsed in water and mounted. Negative controls with a primary polyclonal rabbit antibody against GFP, a non-human protein, showed no staining. All stainings with respective antibodies were conducted at the same time, under the same conditions. The intensity of the staining of the tumour was compared with that of the corresponding normal tissue and graded as follows: 0=no staining, +=less staining than the corresponding kidney cortex from the same patient, ++=equal staining to the kidney cortex tissue, +++=more staining than the kidney cortex.

## RESULTS

### Quantitative RNA analysis

**Figure 1:**
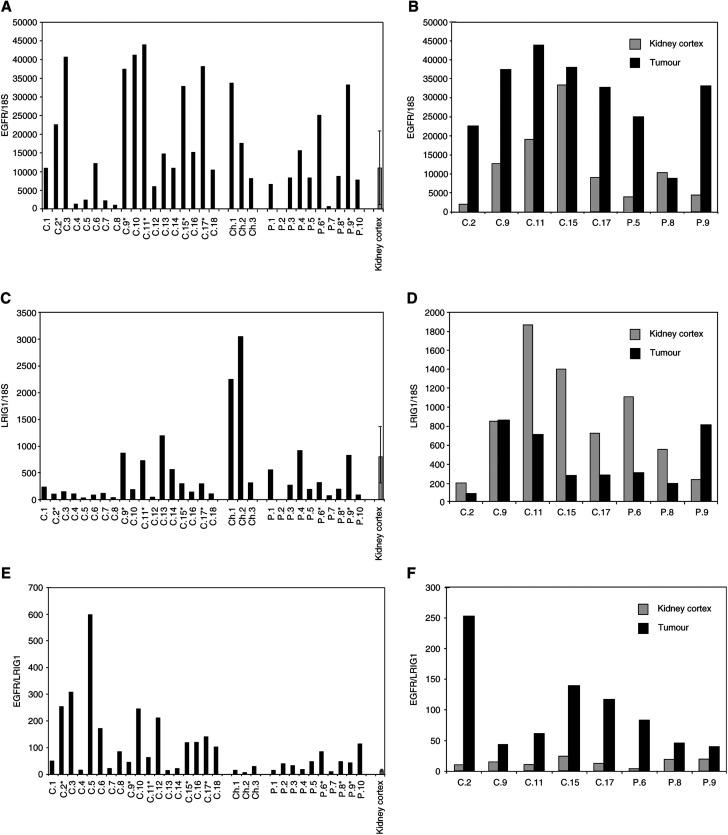
Expression of EGFR and LRIG1 mRNA in RCC and normal kidney. Total RNA was prepared from respective tumour and normal tissues and analysed for EGFR and LRIG1 mRNA and 18S rRNA by quantitative real-time PCR. EGFR and LRIG1 levels were normalised to the 18S rRNA levels in the respective samples. (**A**) EGFR mRNA expression in 31 RCC patients divided by tumour subgroups. (**B**) EGFR mRNA expression in eight RCC tumours and matching normal kidney tissue from the same patients. (**C**) LRIG1 mRNA expression in 31 RCC patients divided by tumour subgroups. (**D**) LRIG1mRNA expression in eight RCC tumours and matching normal kidney tissue from the same patients. (**E**) The EGFR/LRIG1 expression ratio in 31 RCC patients divided by tumour subgroups. (**F**) The EGFR/LRIG1 expression ratio in eight RCC tumours and matching normal kidney tissue from the same patients. Samples C.1–18 were conventional RCCs, samples Ch.1–3 were chromophobic RCCs and samples P.1–10 were papillary RCCs. In (**A**), (**C**) and (**E**), the mean of normal tissues in (**B**), (**D**) and (**F**), respectively, and an extra normal sample (from a patient with a non-RCC tumour) are displayed with the standard deviation indicated by error bars.

In order to examine the mRNA expression of EGFR and LRIG1 in RCC, 31 tumour RNA samples and corresponding kidney tissue in eight of these patients were analysed by quantitative RT–PCR. Expression of the EGFR was observed in all analysed samples. The expression of EGFR was elevated in most of the tumours compared to the mean in the kidney cortex (Figure 1A

) and was markedly increased in six of the eight tumours for which the corresponding kidney cortex was available for comparison. One conventional RCC with very high EGFR expression in the normal tissue and one papillary tumour did not show any significant increase in the tumour tissue (Figure 1B). LRIG1 expression appeared downregulated in the majority of tumours (Figure 1C) and was decreased in six of eight tumours compared with kidney cortex tissue from the same patient. One conventional and one papillary RCC showed unchanged or increased LRIG1 levels compared to normal kidney tissue (Figure 1D). When relating the LRIG1 and EGFR levels in tumour and kidney to each other, the ratio of EGFR/LRIG1 appeared high, especially in conventional RCCs (Figure 1E). It was increased at least 2.5-fold, in eight out of eight tumours compared to corresponding kidney cortex tissue (Figure 1F), and when comparing the ratio in all tumours with the mean normal value, 21 out of 31 tumours showed at least a two-fold increased ratio.

### Immunohistochemistry

All normal tissue and tumours stained strongly for EGFR (Table 3

**Table 3:** Immunohistochemical staining intensity of EGFR and LRIG1 in tumours compared with normal tissue from the same patients

**Patient**	**Type of RCC**	**EGFR staining**	**LRIG1 staining**
C.2	Conventional	+++	++
C.9	Conventional	++	+^a^
C.11	Conventional	+++	0
C.15	Conventional	++	+
C.17	Conventional	+	+
P.6	Papillary	+++	+^a^
P.8	Papillary	+++	++
P.9	Papillary	++	+

The intensity was graded 0=no staining, +=less staining than the kidney cortex from the same patient, ++=equal staining as kidney cortex, +++=more staining than the kidney cortex.

a Exhibited scattered clusters of positive cells.

, Figure 2

**Figure 2:**
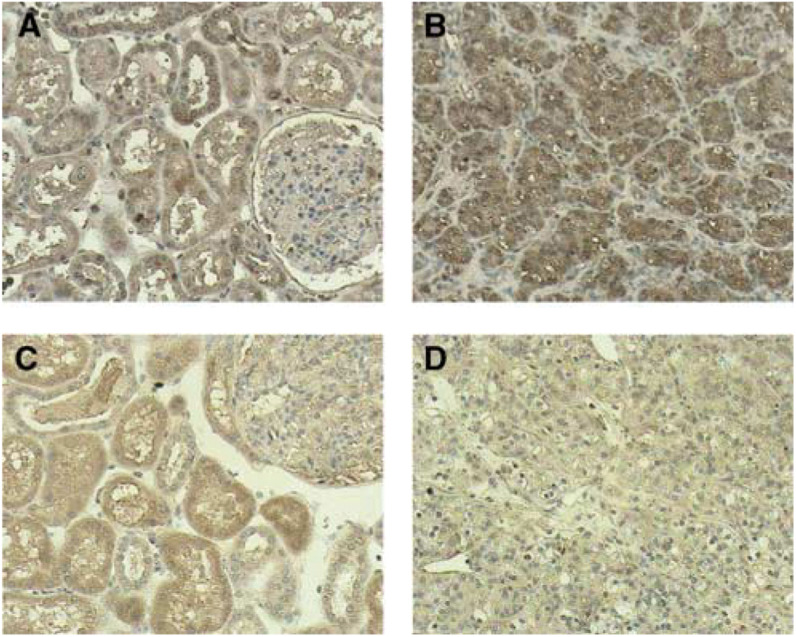
Immunohistochemical staining for EGFR and LRIG1.Tissue sections were stained using indicated antibodies and HRP-conjugated secondary antibodies and counter-stained with Mayer's solution. Samples were obtained from the patient referred to as C.9 and the respective scorings are indicated in parentheses. (**A**) EGFR in normal kidney. (**B**) EGFR in conventional RCC (++). (**C**) LRG1 in normal kidney. (**D**) LRIG1 in conventional RCC (+). Original magnification × 200.

). EGFR in the kidney cortex displayed a pattern with staining of tubular structures, distal tubules staining slightly more intensely than proximal tubules. No staining was seen in connective tissue and in the glomeruli. Tumour cells were homogeneously stained (Figure 2). The staining pattern of LRIG1 in the kidney cortex was very similar to that of EGFR with a most prominent staining in the tubuli (Figure 2), few scattered LRIG1-positive cells were seen in the glomeruli. In tumours, the intensity of LRIG1 staining was generally less pronounced than in the kidney cortex tissue. Some tumours had scattered clusters of positive cells (Table 3).

### Clinical observations

**Figure 3:**
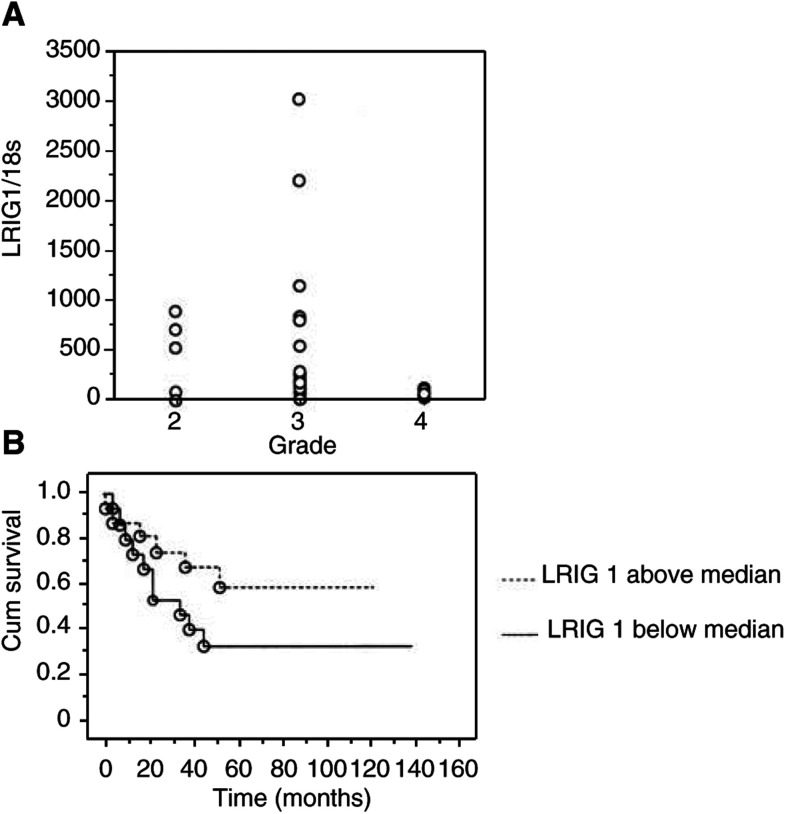
LRIG1 expression in relation to histological grade and survival. LRIG1 mRNA expression levels were quantified using real-time RT–PCR, and normalised to the 18 s rRNA levels in the respective samples. (**A**) Relationship between tumour grade and the expression of LRIG1 mRNA. A tendency of lower expression in grade 4 tumours is seen. Differences between tumours of different grades were not statistically significant (*P*-value of 0.0881, Kruskal–Wallis). (**B**) Kaplan–Meier survival curves for 31 patients with tumours expressing above and below median LRIG1 mRNA levels. Differences between the curves were not statistically significant (*P*-value=0.1499).

Even though the number of patients in this study was limited to 31 patients, we wanted to investigate if any correlations of interest could be seen between the expression of LRIG1 and EGFR and clinical parameters. The most substantial finding was the relation between tumour expression of LRIG1 mRNA and tumour grade (Figure 3A

). The grade 4 tumours expressed lower levels of LRIG1 than seen in grade 2 and 3 tumours, but the groups did not differ significantly (*P*-value 0.0881, Kruskal – Wallis). When divided into groups of high- and low-expressing tumours (median used as cutoff level), a Kaplan – Meier analysis showed a tendency for a nonsignificant survival benefit for patients with high LRIG1-expressing tumours (Figure 3B).

## DISCUSSION

In this study, using quantitative PCR and immunohistochemistry, it was shown that the expression of LRIG1 was decreased and the ratio of EGFR/LRIG1 was increased in tumours *vs* normal tissue. This novel finding in a human tumour specimen lends support to the suggestion that LRIG1 acts as a tumour suppressor and negative regulator of EGFR (Nilsson *et al*, 2001; Hedman *et al*, 2002). EGFR mediates signals that stimulate proliferation, migration and metastasis in many types of tumours, including RCC (Mydlo *et al*, 1989; Ljungberg *et al*, 1994; Yarden, 2001). The localisation of LRIG1 at 3p14.3 is of particular interest since this region is often lost in RCC, particularly in conventional RCC (van den Berg and Buys, 1997). The region of 3p12–14 thus seems important in the search for tumour suppressor genes in RCC. This region has displayed a tumour-suppressing effect when reintroduced into RCC cell lines (Sanchez *et al*, 1994). Earlier, two other possible tumour suppressor genes have been suggested in this area, one at 3p12 and FIT at 3p14.2 (Lovell *et al*, 1999; Velickovic *et al*, 1999). We now suggest that LRIG1 also should be considered as a possible tumour suppressor gene in the genomic region 3p12–14.

Conventional RCC obviously displayed lower expressions of LRIG1 than the kidney cortex, six of eight matched tumour samples had decreased expression and one was unchanged compared to kidney cortex tissue. In contrast, a relatively high expression of LRIG1 was seen in all three chromophobe RCC analysed. When we compared the ratio between EGFR and LRIG1 mRNA, an interesting relationship was observed. The EGFR/LRIG1 ratio was increased at least 2.5-fold in all tumours matched with the corresponding kidney cortex. These observations were seemingly supported by the immunohistochemical analysis. This relationship was not seen when we compared LRIG1 to the other members of the EGFR family (unpublished observations). The increased EGFR/LRIG1 ratio found in RCC lends support to the suggestion that LRIG1 functions as a tumour suppressor and inhibitor of EGFR in humans. Interestingly, we have recently detected the downregulation of LRIG1 in some tumour cell lines of different origin (Hedman *et al*, 2002). Our data demonstrated an apparent difference in the expression of LRIG1 in chromophobe RCC, which appeared to express more LRIG1 than conventional or papillary RCC. This is consistent with the earlier observation that chromophobe RCCs display distinct genetic alterations (Kovacs *et al*, 1997) and expression profiles distinct from conventional RCCs (Young *et al*, 2001). These findings taken together highlight that the different subgroups of RCC should be regarded as different diseases. Although the numbers of tumours analysed were limited, the observations that the tumours of grade 4 seemed to express less LRIG1 than tumours of lower grades and that a small survival benefit for patients with tumours with high expression of LRIG1 were indicated; this might lend further support to the fact that LRIG1 participates in the oncogenesis of RCC.

In conclusion, the underexpression of LRIG1 and the increase in EGFR/LRIG1 ratios found in RCC compared to the kidney cortex indicate that LRIG1 might be a tumour suppressor that counteracts the tumour-promoting function of EGFR. Thus, further studies evaluating larger tumour materials and addressing the molecular function of LRIG1 are warranted.
