# LRIG1 and epidermal growth factor receptor in renal cell carcinoma: a quantitative RT–RCR and immunohistochemical analysis

**DOI:** 10.1038/sj.bjc.6601773

**Published:** 2004-06-08

**Authors:** M Thomasson, H Hedman, D Guo, B Ljungberg, R Henriksson

**Correction to:** *British Journal of Cancer* (2003) **89**, 1285–1289. doi:10.1038/sj.bjc.6601208

A couple of errors have been noted in Table 2

**Table 2:** Oligonucleotide primer and probe sequences for real-time quantitative RT-PCR

**Templates**	**Oligonucleotides**	**Sequences**	**PCR product size (bp)**
EGFR	Forward primer	5′-TCCCTCAGCCACCCATATGTAC-3′	125
	Reverse primer	5′-GTCTCGGGCCATTTTGGAGAATTC-3′	
	Probe	5′-^a^fATCAAC^b^QCACGGAACTTTGGGCGACTATCTGCGTC-3′	
LRIGI	Forward primer	5′-GGTGAGCCTGGCCTTATGTGAATA-3′	108
	Reverse primer	5′-CACCACCATCCTGCACCTCC-3′	
	Probe	5′-^a^fACACAT^b^QGGTAGCGGCCCTCGTGCCCG-3′	
I8S	Forward primer	5′-CGGCGACGACCCATTCGAAC-3′	99
	Reverse primer	5′-GAATCGAACCCTGATTCCCCGTC-3′	
	Probe	5′-^a^fCCTATCAAC^b^QTTCGATGGTAGTCGCCGTGCC-3′	

Note: ^a^f denotes a 5′-conjugated fluorescein and ^b^Q denotes a T with a conjugated Dark Quencher molecule.

of the above article. The corrected table is published below.

The Publisher would like to apologise for any inconvenience this may have caused.

